# Neonatal presentation of growth hormone deficiency in CHARGE syndrome: the benefit of early treatment on long-term growth

**DOI:** 10.20945/2359-3997000000231

**Published:** 2020-03-30

**Authors:** Carla Costa, Eduarda Coutinho, Rita Santos-Silva, Cíntia Castro-Correia, Manuel Carlos Lemos, Manuel Fontoura

**Affiliations:** 1 Unidade de Endocrinologia e Diabetologia Pediátrica Departamento de Pediatria Centro Hospitalar Universitário de São João Porto Portugal Unidade de Endocrinologia e Diabetologia Pediátrica, Departamento de Pediatria, Centro Hospitalar Universitário de São João, Porto, Portugal; 2 Centro de Investigação em Ciências da Saúde Universidade da Beira Interior Covilhã Portugal CICS-UBI, Centro de Investigação em Ciências da Saúde, Universidade da Beira Interior, Covilhã, Portugal

## Abstract

CHARGE syndrome is a complex disorder involving multiple congenital anomalies and is caused by heterozygous mutations in the *CHD7* gene. Growth retardation is a characteristic finding and about 10% of cases present growth hormone (GH) deficiency. GH treatment of short stature in CHARGE syndrome has shown some benefit, but normal height is rarely attained. We report a girl with CHARGE syndrome due to a *de novo* frameshift mutation in the *CHD7* gene (c.2509_2512delCATT), in whom recurrent hypoglycaemia led to the diagnosis of GH deficiency in the second month of life. Early initiation of treatment with recombinant GH resulted in normal growth over ten years of follow-up. This case is the youngest reported CHARGE patient to be diagnosed and treated for GH deficiency and demonstrates that GH deficiency in CHARGE syndrome may manifest early in life through hypoglycaemia, before growth retardation is noted, and can be successfully treated with recombinant GH.

## INTRODUCTION

CHARGE syndrome is a genetic disorder characterized by a variable combination of congenital anomalies that include Coloboma of the eye, Heart defects, Atresia of the choanae, Retardation of growth and development, Genital hypoplasia and Ear abnormalities (1,2). Heterozygous loss-of-function mutations in the gene encoding chromodomain helicase DNA-binding protein 7 (*CHD7*) are the major cause of CHARGE syndrome (3,4).

Children with CHARGE syndrome usually have normal birth weight and length but have markedly impaired postnatal growth (5). Growth retardation is multifactorial and may include factors such as mechanical feeding problems, choanal atresia, gastro-oesophageal fistula or reflux, as well as cardiac and respiratory problems (5). In addition, about 10% of cases present growth hormone (GH) deficiency (6). Recent reports showed that children with CHARGE syndrome, with and without GH deficiency, benefited from treatment with recombinant GH (7,8). However, in these treated cases, height was still subnormal, and this may have been due to the late initiation of treatment, after growth deceleration had already occurred (7,8).

We report a child with CHARGE syndrome, in whom neonatal hypoglycaemia led to an early diagnosis and treatment of GH deficiency, resulting in long-term normal growth.

## CASE REPORT

### Clinical features

**Figure 1:**
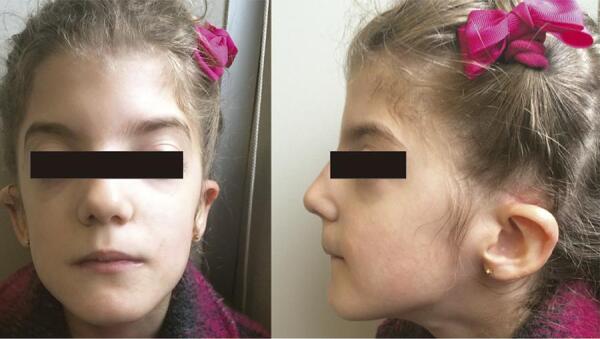
Photographs of the patient at 8 years of age. Dysmorphic features include hypertelorism and bilateral low-set ears.

**Figure 2:**
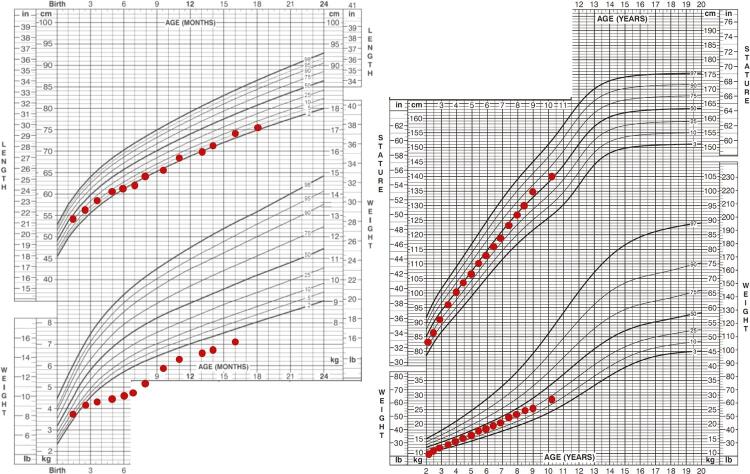
Growth charts of the patient. Left chart refers to length (top) and weight (bottom) in the first two years (adjusted for gestational age) and right chart refers to stature (top) and weight (bottom) from the age of two years onwards.

The patient is the first-born daughter of non-consanguineous Portuguese healthy parents. Pregnancy was complicated by polyhydramnios and emergency caesarean section was performed at 34 weeks of gestation due to prolonged premature rupture of membranes and labour dystocia. Weight, length, and head circumference at birth were 2090 g, 47 cm, and 34 cm, respectively, which were appropriate for gestational age. Apgar scores were 5 at 1 minute and 6 at 5 minutes. She was immediately admitted to an intensive neonatal care unit due to respiratory distress needing mechanical ventilation and suspected choanal and oesophageal atresia. Physical examination during the neonatal period revealed further dysmorphic features, including hypertelorism, bilateral iris coloboma, bilateral low-set ears, right-sided preaxial polydactyly, mild hypotonia, peripheral facial nerve palsy, and a grade 2 systolic heart murmur. At the age of 3 days, the child underwent surgical correction of oesophageal atresia and tracheoesophageal fistula. An echocardiogram revealed a patent ductus arteriosus which was surgically closed at day 30. An endoscopy and computerized tomography (CT) scan confirmed the presence of choanal atresia which was surgically corrected at day 40. A magnetic resonance imaging (MRI) scan showed bilateral hypoplasia of the semicircular canals and bilateral agenesis of the seventh cranial (facial) nerves. Clinical improvement allowed withdrawal of mechanical ventilation at day 44. From day 51, persistent episodes of hypoglycaemia occurred and measurements of serum GH during hypoglycaemia revealed levels of 7.1 ng/mL and 4.7 ng/mL (normal > 10), which indicated a diagnosis of GH deficiency. Serum levels of insulin-like growth factor 1 (IGF-1) were 28 ng/mL and 26 ng/mL (normal 55-324). GH and IGF-1 levels were measured using a solid-phase chemiluminescent enzyme immunometric assay (Immulite 2000, Siemens Healthcare). No other hormonal or metabolic alterations were found. A pituitary MRI was unremarkable. A normal 46,XX karyotype was observed. Treatment with recombinant GH was initiated at day 86. She was discharged from the hospital at the age of 3 months. At the age of 17 months, she underwent surgical correction of right thumb polydactyly. At the age of 4 years, she was fitted with a bilateral hearing aid due to hearing impairment and then with bilateral tympanic ventilation tubes. A divergent strabismus was diagnosed. No olfactory deficits or genitourinary malformations have been noted. The child is presently 10.3 years old and maintains treatment with GH (0.03 mg/kg/day), with a height of 140.9 cm [percentile 52, Standard Deviation Score (SDS) 0.06], a weight of 27.7 kg (percentile 20, SDS -0.85), a height velocity of 5.0 cm/year, and a Tanner stage 1 (Figures 1 and 2). She has a mild cognitive delay with an intelligence quotient (IQ) of 69, and receives occupational, physical, and speech therapy.

### Genetic analysis

**Figure 3:**
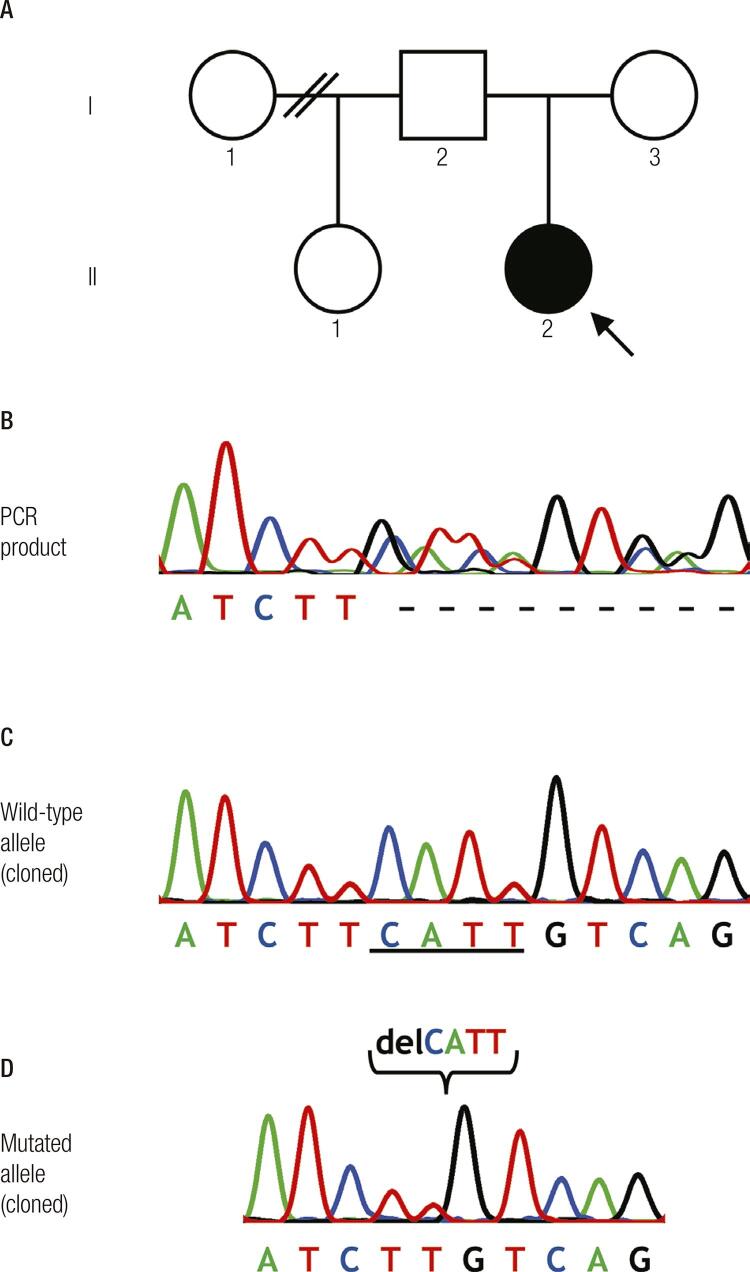
Identification of a CHD7 mutation in the patient. (A) Pedigree of the affected child (arrow). Filled and open symbols represent affected and unaffected individuals, respectively; squares and circles represent males and females, respectively. (B) DNA sequence analysis of the PCR product obtained from the patient revealed a heterozygous deletion of 4 nucleotides (c.2509_2512delCATT) in exon 8. (C) DNA sequence of the normal allele, obtained through pGEM-T cloning of the PCR product from the patient. (D) DNA sequence of the cloned mutated allele, showing a four-base pair (CATT) deletion. Mutation nomenclature was based on the CHD7 complementary DNA (cDNA) reference sequence (GenBank accession number NM_017780), whereby nucleotide c.1 was the A of the ATG-translation initiation codon.

The genetic studies were approved by the local research ethics committee (Faculty of Health Sciences, University of Beira Interior, Ref: CE-FCS-2012-012). Venous blood samples were obtained, after informed consent, from the child and her parents. The patients’ leucocyte deoxyribonucleic acid (DNA) was extracted and used with appropriate polymerase chain reaction (PCR) primers to amplify the 38 exons and their flanking intronic sequences of the *CHD7* gene, utilizing conditions previously described (9). Bi-directional sequencing of the PCR products was performed using the same PCR primers, a CEQ DTCS sequencing kit (Beckman Coulter, Fullerton, CA, USA), and an automated capillary DNA sequencer (GenomeLab TM GeXP, Genetic Analysis System; Beckman Coulter, Fullerton, CA, USA), following the manufacturer’s instructions. This revealed a heterozygous deletion of 4 nucleotides (c.2509_2512delCATT) in exon 8 (Figure 3). This deletion has been previously described (10) and is predicted to result in a frameshift with a premature termination codon at position 842 (H837Vfs*6), instead of the normal termination at codon 2998. The mutation was confirmed by cloning of the PCR products using pGEM-T Easy Vector Systems (Promega Corporation, Madison, WI, USA), followed by DNA sequencing of each allele, as described above. The mutation was not found in the parents DNA, thus it was considered to be a *de novo* mutation.

## DISCUSSION

We describe a child with CHARGE syndrome in whom GH deficiency was diagnosed in the second month of life due to hypoglycaemic episodes. To the best of our knowledge, ours is the youngest CHARGE patient to be diagnosed with GH deficiency and the first in which hypoglycaemia was the clinical presentation of GH deficiency.

GH deficiency has been documented in children with CHARGE syndrome (5-8,11-15). Pinto and cols. (6) performed stimulatory tests for GH secretion in 25 children with CHARGE syndrome, and 3 had low peak GH values consistent with GH deficiency. Other studies found GH deficiency in 1 of 8 patients (12), 3 of 18 patients (11), and 3 of 25 patients (15), respectively. Another study (8) found 15 patients with a low serum GH (<10 ng/mL) in 33 CHARGE patients that had undergone stimulation tests, however, this high incidence was probably due to recruitment bias, since the data were extracted from a GH treatment database. In these reported cases, the diagnosis of GH deficiency was established at several years of age, in the context of growth retardation. Our report of a diagnosis in the second month of life suggests that GH deficiency in CHARGE syndrome can be present at or shortly after birth.

Although GH deficiency is a recognized cause of hypoglycaemia in children (16), so far, there have been no other reports of hypoglycaemia due to GH deficiency in CHARGE syndrome. Our observations illustrate the importance of evaluating GH secretion in children with persistent episodes of hypoglycaemia, which in the case of CHARGE syndrome, could be mistakenly attributed to other aspects of the disorder, such as feeding problems.

There is very limited data in the literature on the effects of GH treatment in children with CHARGE syndrome. Dörr and cols. (8) analysed 51 children who had been treated with recombinant GH and found an increase of median height from –3.6 to –2.2 SDS. However, the median age at the start of GH therapy was 7.6 years and the median duration of GH treatment was only 2.7 years. Thus, long-term data on GH therapy and final height are lacking. In addition, children without proven GH deficiency were included in the study and this may have underestimated the efficacy of GH treatment in true GH-deficient children. Another report by Esposito and cols. (7) described the long-term treatment (from the age of 3 years and 10 months up to adult height) of a CHARGE patient with GH deficiency, with an increase of height from –2.95 to –1.80 SDS. These results suggest that treatment with GH has a positive effect on linear growth, although final height may still be below the 5th percentile.

In contrast to previous reports, our patient maintained a normal growth (percentile 25-50) throughout the 10 years of GH treatment and this could be explained by the early initiation of treatment, prior to the deceleration of growth that typically occurs in children with CHARGE syndrome. However, these results should be viewed with caution as treatment was started when baseline auxological data were still normal and it is difficult to predict what the outcome would have been if treatment had not been offered. Nevertheless, our observations suggest that early recognition of GH deficiency in CHARGE syndrome is important, as treatment with GH may restore normal glucose homeostasis and maintain normal linear growth.

In conclusion, our case report demonstrates that GH deficiency in CHARGE syndrome may manifest early in life through hypoglycaemia, and suggests that early initiation of GH treatment, before growth retardation is noted, may result in long-term normal growth.
